# Access to Healthcare and Medical Expenditure for the Middle-Aged and Elderly: Observations from China

**DOI:** 10.1371/journal.pone.0064589

**Published:** 2013-05-15

**Authors:** Yan Jiang, Yu Wang, Le Zhang, Yang Li, Xiaojun Wang, Shuangge Ma

**Affiliations:** 1 School of Statistics and The Center for Applied Statistics, Renmin University of China, Beijing, China; 2 School of Public Health, Yale University, New Haven, Connecticut, United States of America; Tehran University of Medical Sciences, (Republic of Islamic) Iran

## Abstract

**Background:**

In the evaluation of a healthcare system, it is of interest to identify factors associated with the usage of different healthcare facilities and with different levels of medical expenditure.

**Methods:**

A survey was conducted in January and February of 2012 in China. It focused on the middle-aged and elderly with age of 45 and above. A total of 2,093 people from 1,152 households were surveyed.

**Results:**

For inpatient treatment, the probability of using grade III hospitals, which had the highest level of care, was positively associated with age, being married, living in urban areas, and having higher income. For outpatient treatment, the probability of using grade III hospitals was positively associated with age, being married, working in enterprises, living in urban areas, living in central and western regions, and having higher income, and negatively associated with being farmers. The total and out-of-pocket (OOP) medical expenses were analyzed separately. It was found that the expense level was associated with age, education, occupation, living in urban areas, type of hospital used, insurance being used, and per capita income.

**Conclusion:**

The access to healthcare and level of medical expenditure were found as associated with demographic characteristics. In addition, differences between areas and regions were observed. Such results may be useful for identifying vulnerable population and for tuning future healthcare development policies.

## Introduction

China has the world’s largest population and second largest economy by nominal GDP. Compared with economy, the development of healthcare sector in China has been less impressive [1]. Under the Chinese Healthcare Reform Plan, the healthcare and health insurance system in China is undergoing a system-wide reform, with major objectives including making healthcare more accessible and more affordable [2]. In this study, the focus is on the access to healthcare facilities and medical expenditure incurred by illness conditions.

In the literature, much effort has been devoted to studying the access to and quality of healthcare in China [3], [4], [5], [6]. It is found that in the recent years, significant improvement in healthcare access has happened. Differences in access to care still exist, and multiple factors may have contributed to such differences. Relevant factors may include financial status [3], health insurance status [7], demographic characteristics such as age [8], education and occupation [9], living in urban areas [10], and others. Evaluating the access to healthcare is a multi-layer complicated problem. In this study, we focus on the usage of different hospitals for healthcare. In China, government-run public hospitals are classified into grade I, II and III, and class A and B for each grade. Among them, III-A, III-B and II-A hospitals offer comprehensive healthcare. Grade III hospitals have the capacity of providing healthcare to patients across cities and provinces as well as conducting education and research. In addition, they are in general larger. For example, III-A hospitals are required to have at least 500 beds, while only 400 for III-B and 250 for II-A hospitals. There are also requirements on healthcare providers. For example, at least 20 healthcare providers are expected to have master degrees or higher for an III-A hospital. It is noted that the requirements are much more comprehensive than described above, and the standards and classification of hospitals are still evolving. Compared to grade III, grade II and I hospitals are more regional and smaller, and have lower quality of healthcare. Beyond public hospitals, there are also a small number of private hospitals and other healthcare providing facilities not officially classified as hospitals, complementing public hospitals. Both public and private hospitals are considered in data collection and analysis. Grade III hospitals have the best quality, and the utilization of grade III hospitals can be used as a measure of access to healthcare. Utilization of healthcare facilities has been investigated in [11] and others. However such research has been limited in China. Our literature review suggests that there is no government regulation or guideline on utilizing grade III hospitals. There are a few scattered, not-well-perceived health insurance regulations concerning using a certain facilities for specific diseases. As a major goal of China’s healthcare reform is to make healthcare more accessible to all patients, the study of utilization is of significant interest.

Another important aspect of a healthcare system is expenditure. Two types of expense are of interest, namely gross and out-of-pocket (OOP) expense. Gross medical expense is of interest to government agencies, hospitals, health insurance companies, and others, whereas OOP expense can be more important to patients. The distribution of medical expenditure and associated factors have been studied in a large number of publications [12], [13], [14], [15], [16]. Factors identified to be associated with the level of medical expenditure include demographic characteristics (such as age, gender, education and occupation), insurance status, living in urban, region, and others.

In this study, access to healthcare and medical expenditure are investigated by analyzing data from a recent survey. The overall strategy is similar to that in multiple published studies. On the other hand, this study may differ from the published ones along the following aspects. First, when studying access to healthcare, it focuses on the utilization of grade III hospitals. This aspect has not been carefully investigated in the literature. Second, to more comprehensively describe illness conditions, inpatient, outpatient, and self-treatments are analyzed separately. The three types of treatments correspond to different diseases, incur different levels of expense, and lead to the pursuit of different healthcare strategies. Third, this study focuses on the middle-aged and elderly with age 45 and above. Fourth, data has been generated in a recent survey. With fast economic growth and system-wide healthcare reform, observations made in previous studies not necessarily hold. Given the above considerations, this study is warranted beyond the published ones.

## Methods

### Data Collection

The study protocol was reviewed and approved by the Research Ethics Review Committee at Renmin University of China (RUC). Administration of the study was monitored by the same committee. The in-house survey was conducted in January and February of 2012. Researchers at RUC have been conducting large-scale, longitudinal survey studies on the wellbeing and medical and social security of the middle-aged and elderly. The samples have been randomly collected, covering the majority of municipalities and provinces of mainland China. For the present study, the samples were randomly selected from the samples of existing studies, mainly due to convenience and cost considerations. The samples were from 152 cities of 25 provinces and municipalities of mainland China. The following provinces and municipality were not covered because of resource limitations: Shanghai, Guangxi, Hainan, Xizang, Qinghai and Ningxia. When sampling, stratification by area, region and GDP level of the cities was considered in an attempt to obtain representative samples.

Standard procedures were followed to ensure the high quality of survey and data so generated. All survey staff attended three training sessions and mock interviews. To ensure the quality, the supervisors attended about ten percent of the surveys. At the beginning of each survey, the staff would ask the interviewee to sign a consent form. The written forms were stored at RUC. Basic information would then be collected to determine inclusion. An interviewee would be excluded if he/she had not participated in the existing pension and medical and social security studies conducted by RUC, or was younger than 45, or could not provide reliable information on illness conditions and expense. As all samples were expected to be included in the existing studies, less than ten interviewed subjects were excluded. For the eligible samples, the response rate was 86%.

The survey included both “snapshot” questions (such as age, marital status, education, occupation) and “accumulation” questions (such as income over a period of twelve months prior to survey). Data was collected on (1) demographics and personal characteristics, including gender, age, marital status, education, occupation, household income, personal income, general physical condition, area, and region. Here urban or rural area was defined by “Hukou”, a household registration issued by the central government. The whole China was separated into three geographic regions: eastern, central and western (details available from the authors); (2) type of the nearest hospital, distance from it, and travel time. Such measures may provide information on healthcare availability; (3) information on illness conditions, including the type of treatment (inpatient, outpatient, self-treatment), hospital used, insurance usage, and gross and OOP medical expense. A person was considered ill if he or she was diagnosed by a healthcare professional, experienced discomfort, or was unable to pursue usual activities. Inpatient treatment was defined as an appointment, procedure and/or treatment requiring an overnight stay in a health facility. Outpatient treatment was defined similarly but without an overnight stay. Outpatient treatment included services and medicine administered by a hospital, community health clinic, private health facility, or village health worker. Self-treatment was defined as the scenario where an individual used unprescribed drugs or other medical approaches to treat untreated (and often undiagnosed) medical conditions. For medical expense, data collected included the cost of treatment, medicine and supplies, transportation, food and accommodation, and unofficial gifts (to employees of health facilities, escorts, and caretakers). In addition, lost income (due to illness) was also measured. As argued in [11], lost income is a direct consequence of illness conditions. Although not necessarily a large amount, lost income, nevertheless, should be counted towards total medical expense.

Data were checked on-site and during the input process for obviously unreasonable measurements. Each questionnaire was input by two staff members independently, and then data were cross-checked for accuracy. Data were also checked for internal consistency. For example, the total medical cost could be computed by summarizing the cost of inpatient, outpatient, and self-treatments. In addition, there was also a separate question in the survey asking for the total cost. The two values were compared for quality control.

The questionnaire and sample data are available at http://stat.ruc.edu.cn/a/kexueyanjiu/yanjiujigou/2013/0409/586.html. Per funding regulations, the complete raw data will be publicly available on July 1^st^, 2014. Prior to that date, access to the raw data needs to be applied and approved on a case-by-case basis.

### Data Analysis

Various graphical methods were employed to examine data, and no outlier was identified. Summary statistics were computed for the whole cohort as well as subgroups from different areas and with different medical expense levels. Medical expense was a continuous variable. For presentation simplicity, expense was dichotomized at the median to create two groups, and the low expense group was contrasted against the high expense group. For different types of treatment separately, utilization of grade III hospitals and medical expense were contrasted between urban and rural, and between different health insurance usage statuses (“use insurance” versus “not use insurance”). Multivariate analysis was then conducted, accounting for the joint effects of multiple factors. In the analysis of access, for inpatient and outpatient separately, a binary variable was created, indicating whether a grade III hospital was used for treatment. Logistic regression was conducted, and odds ratios and their significance levels were computed. To be comprehensive, the corresponding univariate logistic regression results were also reported. In the analysis of expense, multivariate linear regression was conducted, and the estimated regression coefficients and their significance levels were computed. Two sets of analyses were conducted. In the first set of analysis, the gross expense of inpatient, outpatient, and self-treatment was separately regressed on patients’ and illness’ characteristics. The second set of analysis was focused on the OOP expense. Model diagnostics was conducted, and no serious deviation from the model conditions was observed. Analysis was conducted using S-Plus Version 8.2 (TIBCO Software Inc.).

Some surveyed subjects had no illness condition in the twelve months period. In our analysis, some of the summary statistics (the first three columns of Table 1) were computed for all surveyed subjects. Other analyses were concentrated only on those with illness conditions. As in most surveys, missingness in data occurred [17]. The simple complete-record-only approach was used to accommodate missingness. As different sets of analyses used different variables, the numbers of records used in analysis were slightly different across the tables.

**Table 1:** Summary statistics for all samples and subgroups from different areas or with different medical expense levels.

		Area		Inpatient treatment cost		Outpatient treatment cost		Self-treatment cost	
	All (n = 2093)	Urban (n = 1268)	Rural (n = 823)	Low (n = 178)	High (n = 152)	Low (n = 605)	High (n = 587)	Low (n = 660)	High (n = 541)
**Gender**									
Male	1053	665	388	93	70	305	270	327	259
Female	1038	603	435	85	81	300	316	332	282
**Ag (mean±sd )**	57.0±10.4	56.5±10.4	57.7±10.4	62.9±11.8	62.4±12.2	56.8±10.0	59.3±11.2	56.3±10.0	58.4±10.8
**Age group**									
45–50	793	501	292	37	40	224	187	262	172
51–60	657	413	244	44	34	197	163	209	178
61–70	305	141	164	38	29	95	110	93	84
>70	298	189	109	56	48	78	119	79	98
**Marital status**									
Single/Divorced/Widowed	204	118	86	26	18	50	70	63	60
Married	1886	1149	737	152	134	553	517	597	481
**Education**									
No schooling	181	43	138	18	21	52	58	64	50
Primary	348	89	259	44	41	113	101	137	97
Junior high	524	254	270	39	38	167	137	163	128
Senior high	413	278	135	36	24	115	116	138	103
Junior college and more	615	597	18	40	27	155	172	156	162
**Occupation**									
Governments	361	355	6	23	15	99	92	85	92
Enterprises	361	303	58	17	18	86	100	117	98
Farmers	430	20	410	30	34	159	104	183	89
Small private business	144	89	55	6	5	35	28	44	44
Others	173	68	105	10	15	50	34	60	42
Retired	376	333	43	63	42	100	154	93	100
Unemployed	217	81	136	25	23	66	67	71	68
**Distance to the nearest hospital (meter)**									
< = 1000	1544	971	573	146	109	449	443	488	393
1001–5000	509	289	220	31	37	152	127	164	137
> = 5001	36	8	28	1	6	4	14	8	9
**Type of the nearest hospital**									
Grade I hospital	904	402	502	64	69	289	275	322	227
Grade II hospital	384	322	62	41	23	106	92	112	107
Grade III hospital	454	373	81	34	36	105	133	112	113
Private hospital	199	116	83	22	16	53	54	46	61
Others	147	53	94	16	8	51	32	68	31
**Travel time to the nearest hospital (minutes)**									
< = 5	953	578	375	78	58	289	241	294	229
6–10	663	439	224	58	55	179	197	211	170
11–30	454	243	211	37	38	126	144	145	137
> = 31	17	5	12	5	0	8	3	9	4
**Region**									
Eastern	1059	642	417	74	94	327	333	358	249
Central	515	306	209	36	27	117	110	123	129
Western	518	320	198	68	31	161	144	179	163
**Areas**									
Rural	824	–	–	65	70	278	218	329	204
Urban	1268	–	–	112	82	326	369	331	336
**Household income (1 K RMB): mean±sd**	94.1±155.1	116.9±187.2	57.8±66.7	100.5±260.7	72.8±63.4	85.5±145.3	106.4±176.7	83.9±100.4	97.5±141.8
**Per capita income (1 K RMB): mean±sd**	31.9±53.7	41.4±64.9	16.8±19.9	34.8±86.4	25.7±24.2	28.8±54.8	35.4±57.4	27.5±34.8	33.7±59.5
**Physical condition**									
Healthy	882	546	336	29	16	250	140	305	159
Just so-so	829	513	316	69	51	257	277	278	237
Slightly sick	238	124	114	44	40	64	99	53	89
Sick	103	58	45	27	29	24	54	17	43
Seriously sick	37	24	13	9	16	10	17	7	13

## Results

### Sample Characteristics

711 urban households with 2,071 members and 441 rural households with 1,510 members were interviewed. Households with no members at or over 45 were discarded. There were a total of 2,093 valid samples, among which 1,268 and 823 lived in urban and rural areas respectively. There were 1,059, 515, and 518 samples in the eastern, central, and western regions, respectively. During a period of twelve months, there were 372, 1,294 and 1,545 observations of inpatient, outpatient and self-treatments.

Table 1 showed that in the whole cohort, there were about an equal number of male and female subjects. There were more females living in the rural areas and having high medical expense, although the gender differences were not big. The age distributions for different areas and different medical expense groups were similar. Similar observations held for other demographic variables including marital status, education, and occupation. For the whole cohort, the per capita income was 31.9 K RMB. A significant rural-urban difference was observed. The average per capita income also differed between different medical expense groups. For 73.9% of surveyed samples, the distance to the nearest hospital was less than 1 KM, while for 1.7%, it was greater than 5 KM. For 83.5% of the samples, public hospitals were closer than private hospitals and other healthcare facilities, with grade I, II and III hospitals accounting for 43.3%, 18.4%, and 21.7% of the observations, respectively. For 45.7% of the samples, the travel time to the nearest hospital was less than five minutes. For 0.8%, the travel time was more than 30 minutes. Although the nearest hospital was not necessarily the one used for care, the distance/time to the nearest hospital, as argued in [11], may still provide useful information on the access to healthcare. Similar problems had been investigated in [18] and others. Table 1 suggests that overall healthcare is accessible. Subgroup analysis led to similar findings, although differences across different subgroups were observed. 42.2% of the samples classified their physical conditions as healthy, whereas 6.7% classified as sick or seriously sick.

For inpatient, outpatient, and self-treatment separately, Table 2 examined the marginal associations between illness characteristics (utilization of hospital, duration of stay, medical expense, cost paid by insurance) and sample characteristics (area, insurance status). For inpatient treatment, urban residents used grade III hospitals the most, whereas rural residents used grade II hospitals the most. Significantly more rural residents used grade I hospitals. Insurance usage was also correlated with utilization of hospitals. The duration of stay was longer for those disease episodes that insurance was used. For inpatient treatment, the average subtotal cost (lost income not included) was 20,515.7 RMB, average lost income was 2,188.7 RMB, and average cost paid by insurance was 13,856.5 RMB. The first finding is that inpatient treatment can be expensive. On average, inpatient treatment cost 8,847.9 RMB, while the per capita income was estimated as 31.9 K RMB. Second, urban-rural differences are observed. For example when insurance was used, the average treatment cost was 23,670.3 RMB for urban, compared to 12,480.3 RMB for rural. Third, insurance status correlates with cost. For urban residents, the average treatment cost was 23,670.3 RMB and 12,040.4 RMB when insurance was used and not used, respectively. For outpatient treatment, grade III and I hospitals were used the most for urban and rural residents, respectively. A significant area difference is observed. Outpatient treatment was much less expensive. Particularly, the average subtotal cost (lost income not included) was 2,438.9 RMB, lost income was 200.4 RMB, and cost paid by insurance was 1,759.7 RMB. The associations between sample and illness characteristics were observed. The distributions of self-treatment were not significantly different for different subgroups. The average treatment cost was 719 RMB, lost income was 140.3 RMB, and amount paid by insurance was 492.5 RMB.

**Table 2:** Summary statistics for subgroups in different areas and with different insurance usage status.

	Urban		Rural		Total
	Use insurance	Not use insurance	Use insurance	Not use insurance	
**Inpatient treatment**					
**Sample size**	197	26	128	21	372
**Type of hospital: count**					
Grade I hospital	18	3	28	1	50
Grade II hospital	51	9	52	8	120
Grade III hospital	117	14	37	7	175
Private hospital	5	0	1	2	8
**Duration(days): mean±sd**	27.0±37.6	14.6±12.8	22.3±33.0	16.9±14.0	24.0±34.0
**Cost(RMB): mean±sd**					
Treatment	23670.3±50331.4	12040.4±15396.5	12480.3±18968.9	12404.8±18304.1	18387.1±39119.0
Medicine/supplies	1231.7±3752.5	1057.7±1666.4	2318.6±17763.9	1809.5±5470.7	1624.3±10816.5
Transportation, food, accommodation	2848.6±9110.4	3328.85±4899.8	2317.42±13280.7	1250.0±1386.7	2609.2±10302.8
Unofficial gifts	350.3±1829.5	750.0±2196.8	572.5±2805.8	170.0±349.6	445.2±2197.1
Subtotal*	26502.5±49992.0	17000.0±15706.5	13831.8±19942.9	9631.0±8092.8	20515.7±38940.4
Lost income	813.4±3239.5	1883.2±4634.7	4209.4±26792.4	2515.0±4637.2	2188.7±16288.3
**Paid by insurance (RMB): mean±sd**	17574.0±40585.9	–	8225.8±21177.6	–	13856.5±34510.2
**Outpatient treatment**					
**Sample size**	418	351	185	340	1294
**Person-times: mean±sd**					
Total	8.3±13.3	4.6±5.5	5.2±5.7	5.1±8.1	6.0±9.5
Grade I hospital	2.3±10.0	1.1±2.5	3.1±4.8	2.1±3.8	2.0±6.4
Grade II hospital	2.0±5.3	1.4±3.6	0.8±2.3	1.1±6.0	1.4±4.8
Grade III hospital	4.2±8.8	1.9±3.6	0.6±2.0	0.8±3.3	2.2±5.8
Private hospital	0.2±1.5	0.4±1.9	0.3±2.1	0.6±2.6	0.39±2.1
**Cost (RMB): mean±sd**					
Treatment	3144.2±5723.9	1450.7±4046.0	912.6±1539.5	1067.1±2267.4	1823.2±4192.3
Medicine/supplies	540.0±1435.5	447.0±1554.0	184.2±389.4	405.3±1718.1	427.2±1456.5
Transportation, food, accommodation	303.8±3299.0	85.6±337.6	114.8±360.5	68.9±165.4	155.5±1885.4
Unofficial gifts	34.2±260.2	39.5±566.7	55.7±401.6	30.1±353.8	37.7±404.2
Subtotal*	4055.8±7749.3	1823.2±3982.3	1343.9±2617.5	1670.8±5514.9	2438.9±5826.1
Lost income	125.2±587.3	73.5±382.3	348.2±1063.1	334.2±1234.8	200.4±858.1
**Paid by insurance (RMB): mean±sd**	2291.7±4362.2	–	690.22±2731.7	–	1759.7±3963.8
**Self-treatment**					
**Sample size**	349	606	76	514	1545
**Person-times: mean±sd**	8.2±23.4	10.7±33.9	6.7±7.4	7.3±9.0	8.8±24.6
**Cost (RMB): mean±sd**					
Subtotal*	875.4±1776.6	846.7±1779.5	476.0±668.1	501.7±1061.6	719.0±1540.2
Lost income	102.9±520.2	118.7±777.4	198.7±729.9	178.8±1133.9	140.3±874.8
**Paid by insurance (RMB): mean±sd**	239.4±413.3	–	548.7±804.9	–	492.5±758.2

Subtotal is the sum of treatment, medicine and supplies, transportation, food, accommodation, and unofficial gifts.

### Utilization of Grade III Hospital

Table 3 suggested that whether a grade III hospital was used for inpatient treatment was significantly associated with age, marital status, area, and per capita income. More specifically, older people were more likely to use grade III hospitals (odds ratio 2.375 for the age group 61–70, and 3.087 for the age group>70). This observation may be partly explained by the fact that older people are more likely to have more serious illness conditions, which demand a higher level of care. Being married was positively associated with using grade III hospitals (odds ratio 2.137). Compared with rural, urban residents were more likely to use grade III hospitals (odds ratio 2.366). Grade III hospitals are mainly located in cities, particularly large cities, which may create access barrier for rural residents. In the literature, we did not find information on the “intended distribution” of grade III hospitals. The observation on rural-urban difference may assist future distribution of healthcare resources. Another significant factor is per capita income (odds ratio 1.012). Because grade III hospitals provide a higher quality of care, getting treated in such hospitals can be more expensive, even after adjusting for insurance payment. Urban residents have higher income. In 2011, the per capita net income of rural residents was 6,977 RMB; In comparison, the median per capita disposable income of urban residents was 19,118 RMB [19]. The higher income of urban residents and higher cost of grade III hospitals can partly explain the observed positive associations for per capita income and area.

**Table 3:** Univariate and multivariate logistic regression analysis of the prevalence of using grade III hospital for inpatient and outpatient treatments.

	Inpatient (N = 372)				Outpatient (N = 1307)			
	N	n (%)	OR (*P*)	aOR (*P*)	N	n (%)	OR (*P*)	aOR (*P*)
**Gender**								
Female	187	85(45.5)	1	1	678	213(31.4)	1	1
Male	184	104(56.5)	1.560(0.033)	1.136(0.608)	628	211(33.6)	1.105(0.400)	0.961(0.788)
**Age**								
45–50	87	44(50.6)	1	1	458	138(30.1)	1	1
51–60	89	39(43.8)	0.762(0.370)	1.054(0.888)	402	139(34.6)	1.226(0.164)	1.478(0.028)
61–70	73	38(52.1)	1.061(0.852)	2.375(0.048)	214	64(29.9)	0.989(0.953)	1.758(0.027)
>70	118	65(55.1)	1.199(0.523)	3.087(0.014)	209	72(34.4)	1.219(0.266)	1.817(0.031)
**Marital status**								
Single/Divorced/Widowed	53	22(41.5)	1	1	139	39(28.1)	1	1
Married	318	167(52.5)	1.558(0.140)	2.137(0.050)	1166	385(33.0)	1.264(0.239)	1.785(0.025)
**Education**								
No schooling	46	18(39.1)	1	1	117	22(18.8)	1	1
Primary	91	39(42.9)	1.167(0.676)	1.015(0.973)	231	42(18.2)	0.960(0.888)	0.832(0.590)
Junior high	85	43(50.6)	1.593(0.211)	1.343(0.534)	332	89(26.8)	1.582(0.086)	1.148(0.681)
Senior high	73	38(52.1)	1.689(0.170)	1.254(0.652)	254	86(33.9)	2.210(0.003)	1.172(0.658)
Junior college and more	74	50(67.6)	3.241(0.003)	1.653(0.376)	364	185(50.8)	4.463(0.000)	1.854(0.103)
**Occupation**								
Governments	43	28(65.1)	1	1	217	96(44.2)	1	1
Enterprises	38	24(63.2)	0.918(0.854)	1.066(0.902)	204	93(45.6)	1.056(0.781)	1.724(0.016)
Farmers	67	23(34.3)	0.280(0.002)	0.994(0.992)	283	22(7.8)	0.106(0.000)	0.458(0.032)
Small private business	14	8(57.1)	0.714(0.592)	0.785(0.748)	72	17(23.6)	0.390(0.002)	0.843(0.640)
Others	29	16(55.2)	0.659(0.397)	1.123(0.859)	94	31(33.0)	0.620(0.065)	1.655(0.137)
Retired	124	66(53.2)	0.610(0.178)	0.501(0.157)	275	120(43.6)	0.976(0.894)	1.510(0.109)
Unemployed	53	23(43.4)	0.411(0.036)	0.952(0.935)	144	37(25.7)	0.436(0.000)	1.250(0.491)
**Areas**								
Rural areas	146	51(34.9)	1	1	533	75(14.1)	1	1
Urban areas	225	138(61.3)	2.955(0.000)	2.366(0.025)	773	349(45.1)	5.026(0.000)	2.088(0.001)
**Regions**								
Eastern	183	96(52.5)	1	1	711	214(30.1)	1	1
Central	82	38(46.3)	0.783(0.358)	0.822(0.523)	273	98(35.9)	1.301(0.080)	1.950(0.000)
Western	107	55(51.4)	0.959(0.862)	1.008(0.979)	323	112(34.7)	1.233(0.142)	1.641(0.004)
**Use insurance**								
No	47	22(46.8)	1	1	694	192(27.7)	1	1
Yes	323	165(51.1)	1.187(0.584)	1.091(0.805)	602	231(38.4)	1.628(0.000)	1.213(0.183)
**Per capita income** **(thousand Yuan)**	–	–	1.016(0.002)	1.012(0.028)	–	–	1.014(0.000)	1.011(0.000)

N: number of subjects; n: number treated at grade III hospitals. OR (P): odds ratio (p-value) from univariate logistic regression; aOR (P): adjusted odds ratio (p-value) from multivariate logistic regression.

For outpatient, multiple factors were found to be associated with using grade III hospitals, including age, marital status, occupation, area, region, and per capita income. Age was found be to positively associated with using grade III hospitals (odds ratios equal to 1.478, 1.758, and 1.817 for the three age groups respectively). The same rationale as for inpatient treatment may hold here. Being married was positively associated with using grade III hospitals. Unlike with inpatient, occupation was found to be significant. In particular, with working in governments as baseline, people working in big enterprises were significantly more likely to use grade III hospitals (odds ratio 1.724), while farmers were significantly less likely (odds ratio 0.458). Multiple factors may have contributed to this finding. The first is the correlation between occupation and other factors, particularly income and area. The second is that, as described in [14], people with different occupations usually have different types of insurance, which have different regulations on using certain hospitals. Area was found to be a significant factor (odds ratio for urban 2.088). Similar arguments as for inpatient treatment may hold here. Regional differences were observed, with central (odds ratio 1.950) and western (odds ratio 1.641) more likely to use grade III hospitals than eastern. The regional difference in health resource allocation and correspondingly access has been noted in publications [20]. However, this study may be among the first to note the regional difference for outpatient treatment in China. Like for inpatient, per capita income was positively associated with using grade III hospitals (odds ratio 1.011 for 1 K RMB).

### Medical Expenditure

In the first set of analysis, for inpatient, only the type of hospital was found to be significant (estimated regression coefficient 17,159.7 RMB, with grade I hospital as baseline). The higher cost of grade III hospitals has been previously noted and is reasonable. In addition, two education levels were found to be borderline significant (primary school: estimated regression coefficient −16,683.4 RMB, p-value 0.055; junior college and more: estimated regression coefficient −20,932.7 RMB, p-value 0.063). For outpatient, the education level “primary school” was borderline significant (estimate 1,463.0 RMB, p-value 0.096). Occupation was found to be significant. With governments as baseline, retired had significantly higher cost (estimated regression coefficient 3,245.2 RMB), and “no job” also had higher cost (estimated regression coefficient 2,444.2 RMB). In addition, the “others” category was borderline significant (estimated regression coefficient 2,024.5 RMB, p-value 0.057). The type of hospital used was significant, with grade II (estimated coefficient 1,119.7 RMB) and III (estimated coefficient 2,538.3 RMB) hospitals cost more than grade I hospitals. Using health insurance was positively associated with cost (estimated coefficient 1,219.3 RMB). This association may have an indirect interpretation. As has been noted in published studies [11], the decision to use insurance was associated with demographic characteristics (such as income and education), type of hospital used, and illness characteristics. Per capita income was found to be borderline significant (p-value 0.067). For self-treatment, age was found to be significant. More specifically, the estimated cost increased with age (estimates 272.1 RMB, 574.3 RMB, and 960.2 RMB, respectively). Another significant factor was occupation. With governments as baseline, (borderline) significant levels included enterprises (estimate −09.4 RMB, p-value 0.05), farmers (estimate −347.6 RMB, p-value 0.098), and retired (estimate −477.3 RMB, p-value 0.011). Living in urban areas was associated with more cost (estimate 380.4 RMB). The positive association for per capita income was borderline significant.

In the analysis of OOP expense, for inpatient, the findings were similar to those with the total expense. That is, only using grade III hospital was borderline significant (estimate 10,329.8 RMB, p-value 0.061). It is noted that, this estimate and several others (for example those for age groups) had considerably large estimates. Because of the high variation of expense, their estimates were not statistically significant. However, estimates of such magnitudes may deserve further attention. For outpatient, the findings were also similar to those with inpatient. Significant factors included education, occupation, and using grade II and III hospitals. However, using health insurance and per capita income were no longer significant. For self-treatment, significant factors included age, occupation, and area. The association for per capita income was no longer significant.

## Discussion and Conclusion

### Limitations

Data were collected from questionnaires only. Internal cross-check was conducted for quality control. However, there was a lack of external cross-check (for example using insurance reimbursement data). Collecting additional data was not feasible with limited resources. It is noted that quite a few published studies may share the same limitation. The survey was designed to collect information for a period of twelve months. For an individual, illness conditions, particularly inpatient treatment, may vary from year to year. Even though it might be possible to collect information for a longer period, such an effort was not pursued because of the concern on recall bias. The survey collected cross-sectional, observational data. With such data, only associations, as opposed to causality, could be inferred. Many published studies, such as [11], [14] and others, share this limitation. Data was collected using in-house surveys. The nature of survey inevitably led to certain drawbacks, including limited information, possible recall bias, and others [21]. This study collected information on inpatient, outpatient, and self-treatment separately, and can be more informative than studies that collect illness and cost information as a whole. Detailed information on disease was not available. Thus some of the analysis results should be interpreted with cautions. Even though grade III hospitals have the highest quality of care, for less serious diseases, utilizing such hospitals is not necessarily desirable. The goal of our study is to provide an objective description of the utilization status. It is beyond our scope to determine whether such utilization is justifiable. The surveyed samples were selected in a random manner. China is a huge country with significant differences across areas and regions. With 2,093 samples from 25 provinces and municipalities, this study may have limited power to provide an accurate account of the whole Chinese population. All samples were 45 years old or above. Although this cohort is of significant interest, particularly considering the growing aging population, the younger cohort may deserve attention in future studies.

## Discussion

Access is an important measure in the evaluation of effectiveness of a healthcare system, especially considering that a major goal of China’s health reform is to make healthcare more accessible. Quantifying access is a complicated problem. In this survey, access was measured by the type of hospital used for treatment as well as characteristics of the nearest hospital. Regression analysis was conducted on whether a grade III hospital was used. This strategy has been motivated by [11] and references therein. As discussed in the Background section, the access to healthcare has been studied in a large number of publications. Findings in this study partially match those in the literature. More specifically, financial status, age, occupation, and living in urban areas were found to be significant, as in published studies. However, insurance, education, and gender were found to be not significant, which differs from some of the published studies [22]. Multiple factors may have contributed to the differences. For example, the healthcare and health insurance systems in China differ significantly from those in other countries. The survey has been focused on the population with age 45 and above. And with a fast economic development and healthcare system reform, the experiences of Chinese population are also changing.

It has been recognized that the financial consequences of illness conditions can be substantial, particularly in less-developed countries [23], [24], [25]. For the 2,093 surveyed samples, there were 372, 1294 and 1545 observations of inpatient, outpatient and self-treatments, respectively, during a period of twelve months. The average OOP cost was estimated to be 8,848 RMB (inpatient), 880 RMB (outpatient), and 367 RMB (self-treatment), respectively. The per capita income was estimated to be 31.9 K RMB, and the average household income was estimated to be 94.1 K RMB. For a middle-aged or elderly person, inpatient treatment may cost about 27.7% of the annual income. Such a high percentage, coupled with moderate per capita income, suggest that the expense from inpatient treatment may affect the basic capacities of individuals and households [11]. Outpatient and self-treatments, although less expensive per episode, can still be expensive as they are much more frequent. Among the measured variables, gender, marital status, and region were found to be not associated with any expense. All other variables were associated with one or more types of expense. As described above, most of the identified associations have intuitive interpretations and match those in published studies. The analysis results in Table 4 also suggest that there are a few other factors with considerably large estimated coefficients. Although not significant, they may deserve attention in future studies.

**Table 4:** Multivariate analysis of medical cost.

	Total cost of inpatienttreatment(n = 323)	Total cost of outpatienttreatment(n = 1063)	Total cost ofself-treatment(n = 1301)	OOP cost ofinpatienttreatment(n = 306)	OOP cost ofoutpatienttreatment(n = 1033)	OOP cost ofself-treatment(n = 1118)
**Gender (Female, reference group)**	616.1 (0.902)	1.9 (0.997)	−122.5 (0.213)	−249.7 (0.947)	48.7 (0.899)	−90.7 (0.398)
**Age group (45–50, reference group)**						
51–60	−6047.7 (0.402)	405.9 (0.431)	272.1 (0.016)	−5629.7 (0.303)	54.2 (0.905)	304.2 (0.015)
61–70	−9331.3 (0.272)	−3.8 (0.996)	574.3 (0.001)	−6547.4 (0.303)	−327.4 (0.593)	651.2 (0.000)
>70	−5606.7 (0.524)	−177.0 (0.827)	960.2 (0.000)	−7869.7 (0.235)	−528.7 (0.458)	938.8 (0.000)
**Marital status (Single/Divorced/Widowed, reference group)**						
Married	6193.1 (0.422)	−229.0 (0.760)	46.5 (0.782)	2480.5 (0.668)	168.8 (0.796)	23.5 (0.896)
**Education (No schooling, reference group)**						
Primary	−16683.4 (0.055)	1463.0 (0.096)	74.7 (0.705)	−4043.3 (0.532)	1563.0 (0.042)	86.4 (0.669)
Junior high	−13042.5 (0.169)	−338.8 (0.711)	163.2 (0.425)	1717.1 (0.807)	−445.1 (0.577)	168.9 (0.425)
Senior high	−15747.8 (0.115)	64.9 (0.948)	139.1 (0.529)	−1111.6 (0.882)	−400.3 (0.644)	122.9 (0.594)
Junior college and more	−20932.7 (0.063)	900.1 (0.405)	80.9 (0.735)	−3934.6 (0.638)	−17.1 (0.986)	145.9 (0.566)
**Occupation (Governments, reference group)**						
Enterprises	−821.6 (0.935)	885.9 (0.223)	−309.4 (0.050)	5451.2 (0.470)	182.3 (0.777)	−301.0 (0.098)
Farmers	1301.3 (0.915)	866.9 (0.370)	−347.6 (0.098)	1464.8 (0.873)	−210.8 (0.804)	−281.0 (0.218)
Small private business	−4948.5 (0.744)	1500.5 (0.159)	71.2 (0.751)	172.6 (0.988)	660.2 (0.481)	143.6 (0.552)
Others	18336.7 (0.158)	2024.5 (0.057)	−286.7 (0.209)	14528.8 (0.137)	1161.5 (0.218)	−178.8 (0.476)
Retired	1940.1 (0.839)	3245.2 (0.000)	−477.3 (0.011)	1923.5 (0.786)	1737.6 (0.015)	−370.9 (0.079)
No jobs	−3810.5 (0.743)	2444.2 (0.015)	−84.8 (0.705)	1106.7 (0.898)	1387.2 (0.113)	19.1 (0.938)
**Areas (Rural areas, reference group)**						
Urban areas	7166.0 (0.350)	−194.5 (0.764)	380.4 (0.009)	−1984.8 (0.738)	−452.4 (0.431)	423.4 (0.006)
**Regions (Eastern, reference group)**						
Central	1293.3 (0.835)	−53.1 (0.924)	117.3 (0.320)	6474.5 (0.172)	−78.8 (0.873)	126.3 (0.333)
Western	−10395.1 (0.062)	−809.1 (0.107)	134.8 (0.226)	−3618.7 (0.383)	−555.3 (0.208)	106.2 (0.381)
**Type of hospital (Grade I hospital, reference group)**						
Grade II hospital	−3424.7 (0.649)	1119.7 (0.051)	–	−1930.1 (0.732)	1157.1 (0.022)	–
Grade III hospital	17159.7 (0.020)	2538.3 (0.000)	–	10329.8 (0.061)	1847.7 (0.000)	–
Private hospital	−8309.2 (0.634)	971.1 (0.268)	–	870.9 (0.946)	479.4 (0.531)	–
**Using health insurance (No, reference group)**						
Yes	8422.9 (0.249)	1219.3 (0.005)	19.2 (0.860)	−1911.9 (0.721)	−216.8 (0.565)	−144.7 (0.288)
**Per capita income** **(1** **K Yuan)**	6.7 (0.857)	6.9 (0.067)	1.7 (0.073)	−13.8 (0.611)	−0.1 (0.969)	1.4 (0.216)

In each cell: estimated regression coefficient (p-value).

### Conclusion

Research on the healthcare system in China has attracted tremendous attention. This study has been focused on the access to healthcare and medical expense. Data from an in-house survey was analyzed. Factors significantly associated with using grade III hospitals for inpatient and outpatient treatments and with medical expense were identified. The majority of the findings are consistent with the literature. However there are new findings which may complement the existing studies and provide additional insights. Despite several limitations, this study may still be valuable. Particularly, the main objectives of China’s healthcare reform include improving access and reducing cost. This study may help identify the subgroups that need the most attention and eventually facilitate developing policy interventions.
